# Neutrophil Mechanosignaling Promotes Integrin Engagement With Endothelial Cells and Motility Within Inflamed Vessels

**DOI:** 10.3389/fimmu.2018.02774

**Published:** 2018-11-28

**Authors:** Vasilios A. Morikis, Scott I. Simon

**Affiliations:** Simon Lab, Department of Biomedical Engineering, University of California, Davis, Davis, CA, United States

**Keywords:** neutrophil recruitment, mechanosignaling, selectin, integrin, outside-in signaling

## Abstract

Neutrophils are the most motile of mammalian cells, a feature that enables them to protect the host against the rapid spread of pathogens from tissue into the circulatory system. A critical process is the recruitment of neutrophils to inflamed endothelium within post-capillary venules. This occurs through cooperation between at least four families of adhesion molecules and G-protein coupled signaling receptors. These adhesion molecules convert the drag force induced by blood flow acting on the cell surface into bond tension that resists detachment. A common feature of selectin-glycoprotein tethering and integrin-ICAM bond formation is the mechanics by which force acting on these specific receptor-ligand pairs influences their longevity, strength, and topographic organization on the plasma membrane. Another distinctly mechanical aspect of neutrophil guidance is the capacity of adhesive bonds to convert external mechanical force into internal biochemical signals through the transmission of force from the outside-in at focal sites of adhesive traction on inflamed endothelium. Within this region of the plasma membrane, we denote the inflammatory synapse, Ca^2+^ release, and intracellular signaling provide directional cues that guide actin assembly and myosin driven motive force. This review provides an overview of how bond formation and outside-in signaling controls neutrophil recruitment and migration relative to the hydrodynamic shear force of blood flow.

## Leukocyte recruitment cascade at sites of inflammation

**Figure 1:**
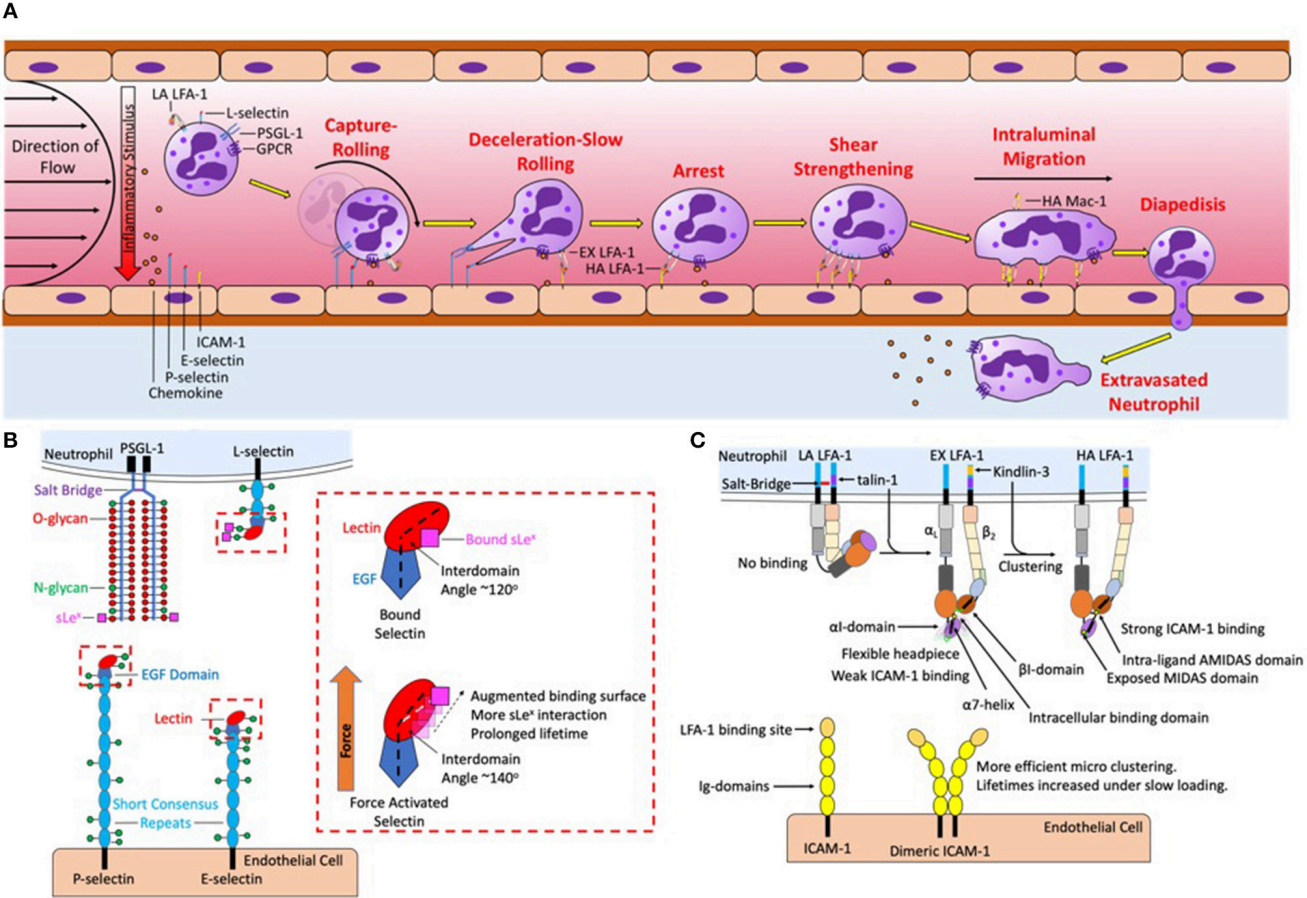
Neutrophil recruitment under shear flow is coordinated by selectins, chemokines, and integrin ligand binding and signaling. **(A)** The sequential steps of neutrophil recruitment under shear flow is initiated by hydrodynamic forces that bring L-selectin and PSGL-1 on the neutrophil surface in contact with P-selectin and E-selectin on the endothelial plasma membrane, mediating capture, and rolling. To resist shear flow selectins and PSGL-1 form transient yet strong bonds that form at the leading-edge to initiate cell rolling and break at the trailing edge under tensile force. Chemokine captured via glycosaminoglycans on the endothelial surface and force acting on L-selectin then activates LFA-1 into an extended conformation to initiate deceleration and slow rolling. A second signal is initiated by L-selectin and CXCR that upshifts integrin into a high affinity state thereby initiating arrest. Initially integrin LFA-1 is spread randomly throughout the surface of the neutrophil, force acting on high affinity LFA-1 initiates their redistribution that reinforces shear resistant cell arrest. Clusters of LFA-1 transduce calcium signaling and neutrophil shape change, leading to high affinity Mac-1 mediated intraluminal crawling along a gradient of chemokine on the endothelial surface, directing the neutrophil to the desired site of migration. Endothelial diapedesis promotes neutrophil access the site of injury or tissue infarct. **(B)** Mechanics of PSGL-1 and L-selectin bond formation play a key role in force transduced signaling. PSGL-1 is primarily decorated with O-glycans while L-selectin is primarily decorated with N-glycans, sLe^x^ deposited on these glycans binds to the lectin domain of P-selectin and E-selectin. Under tensile bond force catch-bonds are formed by a change in intracellular angle of selectin EGF-Lectin domain in all three selectins. Selectin catch-bonds are necessary to mechanosignal activation of LFA-1 under shear conditions in the absence of chemokine. The red box details one potential mechanism in which the selectin interdomain angle between the lectin and EGF domain shifts in response to force allowing for prolonged bond lifetimes. **(C)** LFA-1 is expressed predominantly on membrane microvilli in a low affinity conformation, stabilized by a salt-bridge that clasps together the intracellular α_L_ and β_2_ tails. Signaling through selectin and chemokine initiates talin-1 recruitment to the proximal NPxF motif on the β_2_-integrin that breaks the salt-bridge and extends the integrin, revealing the ICAM-1 binding site in the headpiece. However, the headpiece remains flexible and the MIDAS binding domain remains obscured, supporting weak ICAM-1 bonds. Kindlin-3 binding to the distal NPxF motif promotes transition to a high affinity state and stabilizes microclustering of talin-1 and ICAM-1 bound LFA-1 monomers. Tensile bond force acting on high affinity LFA-1 is transduced intracellularly through a shift in the α_7_ helix of the αI domain. This in turn induces an allosteric shift that exposes the MIDAS domain allowing for recognition of ICAM-1 and stable bond formation. The binding between ICAM-1 and high affinity LFA-1 at low force regimes is amplified by tandem bond formation with dimeric ICAM-1.

Leukocyte recruitment is an evolutionarily conserved process in which the target of natural selection is a fast and efficient immune system that transports neutrophils in numbers appropriate for host defense. A multi-step cascade of adhesive events, which includes ligation and signaling through selectins, integrins, and chemokine receptors, guide neutrophil recruitment to inflamed endothelium (Figure 1A). Adhesive engagement between the neutrophil and the endothelium is initiated by selectins that recognize sialylated and fucosylated carbohydrate ligands expressed on adjacent plasma membranes. E-selectin (CD62E) and P-selectin (CD62P) receptors, upregulated on inflamed endothelium, and L-selectin (CD62L), constitutively expressed on the leukocyte, are strategically positioned on the plasma membrane to form bonds that initiate cell tethering and rolling under the hydrodynamic shear exerted by flowing blood (Figure 1B). Fluid drag forces are resisted by selectin bond tension, which rapidly induces receptor redistribution and formation of focal clusters within the site of adhesive contact between adjacent plasma membranes. For instance, E-selectin and P-selectin binding to sialyl-Lewis^x^ (sLe^x^) on PSGL-1, or E-selectin recognition of sLe^x^ on L-selectin (only on human neutrophils), promotes neutrophil tethering and rolling that induces subsequent selectin interactions such as with glycolipids. Cell rolling allows interrogation of the vascular surface, and at optimum site density between selectins and their ligands a second event occurs that involves intracellular signaling, a process necessary to activate β_2_-integrins. In the absence of high affinity activation of β_2_-integrins, shear resistant adhesion or cell arrest is not observed; a requisite step to initiate neutrophil spreading, polarization, and transendothelial migration (1–4). The importance of β_2_-integrin expression and activation in innate immune function is evident in patients suffering from leukocyte adhesion deficiency 1 (LAD-I), where CD18 expression on the cell surface is lost or reduced, resulting in chronic infections, impaired wound healing, and a defect in neutrophil recruitment (5–7).

The first β_2_-integrin discovered, LFA-1 (also known as CD11a/CD18 or α_L_β_2_), is a key integrin involved in early signaling. LFA-1 converts drag forces of flowing blood into bond tension that transduces intracellular chemical signals. Once bound, high affinity LFA-1 not only acts as an adhesive anchor but also functions as a mechanosensitive receptor capable of transducing external force into internal chemical signals (8–11). The conversion of mechanical force to chemical signals at the site of contact (e.g., inflammatory synapse) can be considered a mechanism of tactile sensing through selectin and integrin bond force transduction that determines through molecular recognition where and when neutrophil emigration occurs (Figure 1C). A cooperative mechanism underlies early activation of LFA-1 that is initiated by selectin mediated capture and rolling, which facilitates G-protein coupled receptor (GPCR) binding of chemokines presented on the glycocalyx of inflamed endothelium. Rolling on E-selectin initiates the rapid extension of LFA-1 that effects deceleration of neutrophils through interaction with its endothelial ligand intracellular adhesion molecule 1 (ICAM-1) (9, 12–14). Chemokine binding of CXCR1 and CXCR2 is sufficient to initiate so called inside-out activation of β_2_-integrins that corresponds with a shift of LFA-1 to a high affinity conformation and promotion of tight bond formation with ICAM-1. Superposition of selectin ligand outside-in signaling during rolling via E-selectin effectively amplifies GPCR inside-out signaling, such that very low levels of chemokine engagement become stimulatory at concentrations that independently do not elicit measurable calcium flux or β_2_-integrin activation (7, 14–16). For example, stimulating neutrophils rolling on E-selectin under shear at a concentration of 0.05 nM IL-8, corresponding to ligation of ~10–100 CXCR receptors per cell, activates a similar level of Ca^2+^ release and up-shift of β_2_-integrin receptors to high affinity as does stimulation of cell suspensions with 5 nM IL-8 under static or very low shear conditions (9, 15). Thus, combined selectin ligand outside-in signaling via E-selectin recognition of sLe^x^ on L-selectin and LFA-1/ICAM-1 bonds effectively amplify signaling via CXCR1/2 by ~100-fold and induces inside-out activation of β_2_-integrin. Ligation of CXCR1 and CXCR2 also activates Mac-1 (also known as CD11b/CD18 or α_M_β_2_) on the plasma membrane (17, 18). While LFA-1 binds the ICAM family of proteins and regulates adhesive events within seconds of cell capture and rolling, Mac-1 recognizes a wide variety of ligands including complement iC3b, fibrinogen, and fibronectin, which facilitates intravascular crawling during paracellular and transcellular migration across inflamed endothelium (19, 20). Force acting on high affinity LFA-1 induces intracellular protein assembly that provides a physical linkage between calcium release-activated channels and calcium stores associated with the endoplasmic reticulum (ER), (21–24). We propose that local regulation of intracellular calcium serves as a secondary messenger downstream of GPCR signaling to regulate neutrophil shape change during the transition from rolling to arrest. While GPCR triggers PLC-β activation of IP3 to elicit calcium release from ER stores on the order of 500 nM of Ca^2+^, to achieve the maximum burst in cytosolic Ca^2+^ flux (~1.0 μM) requires integrin activation, ligation to ICAM-1, and force transduced outside-in signaling (16, 25). In this manner, intracellular Ca^2+^ release functions as a gatekeeper in regulating the conversion of a passive neutrophil in circulation to one that is firmly arrested and poised to transmigrate at sites of vascular inflammation expressing appropriate levels of chemokine agonist, E-selectin, and ICAM-1.

## Mechanosignaling via selectins promotes LFA-1 activation

Selectin and integrin receptors are by nature's design mechanically tuned to function as de facto tactile sensors that convert the drag forces of flowing blood to tensile bond force that transduces biochemical signals at sites of focal adhesion. Mechanosignaling superposes with chemokine signaling to provide for precise spatiotemporal regulation of neutrophil recruitment at vascular sites proximal to tissue insult and injury. Inactive LFA-1 exists in a compact bent conformation with close association of the α and β extracellular domains that maintain a low binding affinity for ICAM-1 (Figure 1C). Rolling on E-selectin and P-selectin in the absence of chemokine induces extension of LFA-1 into an intermediate affinity conformation that supports slow neutrophil rolling at velocities of ~5 μm/s. In the extended conformation the extracellular domain of LFA-1 swings outward, but remains in a relatively closed state, such that bulkier ligands are sterically hindered from accessing the metal ion-dependent adhesion site (MIDAS) domain associated with high affinity binding (Figure 1C) (12, 26–28). This physiological mechanism for neutrophil deceleration is observed in a parallel plate flow chamber whereby the dynamics of LFA-1 extension is reported by increased binding of antibody extension reporter, KIM127 (29, 30). Inside-out stimulation elicits the release of a salt bridge between the α and β chains in the intracellular component of the integrin that triggers opening of the extracellular domain in a switchblade-like motion thereby exposing the MIDAS ligand binding domain (Figure 1C) (28). Increased binding affinity corresponds to a decrease in the k_off_, the rate constant for dissociation of the complex, which coincides with formation of LFA-1 bond clusters with ICAM-1 that supports shear resistant cell arrest under flow conditions (12, 31, 32). Blocking high affinity LFA-1 using a small molecule allosteric agonist that prevents the MIDAS domain from opening elicits suppression of cell arrest, while slow rolling via the extended conformation is maintained (12, 33). The increase in association rate and maintenance of slow rolling under conditions whereby the high affinity state is blocked implicates LFA-1 extension as the braking mechanism that neutrophils utilize to initiate the transition to firm arrest. Data supports the contention that intermediate-affinity of LFA-1 can participate in neutrophil capture and rolling; however, further up-regulation to high affinity by other activation mechanisms, including selectins, chemokines, and inflammatory lipids, is critical for the efficient transition to arrest and prompting of transendothelial migration. In particular, selectin ligand outside-in signaling functions to activate β_2_-integrins, elicit Ca^2+^ flux, and promote F-actin formation all of which promote cell polarization and a migratory phenotype (4, 13). In particular, L-selectin and P-selectin glycoprotein ligand 1 (PSGL1) function as mechanosensitive receptors that trigger LFA-1 extension and transition to integrin mediated activation processes.

**Figure 2:**
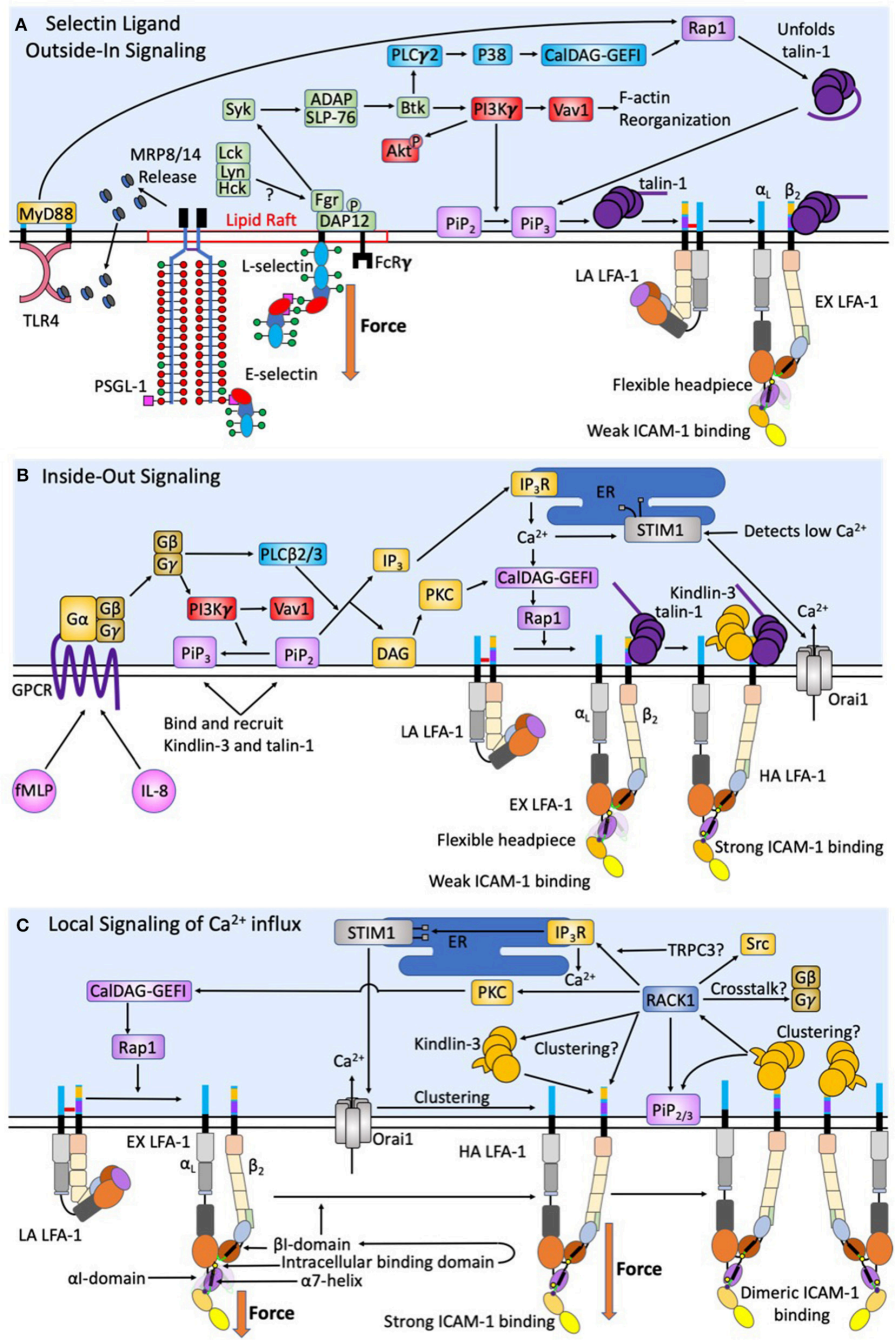
Intracellular signaling events act synergistically to promote human neutrophil arrest and shape change. During initial capture and rolling on inflamed endothelium a low baseline level of intracellular calcium is maintained. **(A)** Force acting on L-selectin and PSGL-1 induces clustering and recruitment of FcRγ and DAP-12 into lipid rafts. Phosphorylation by Fgr results in Syk activation. Other Src family kinases have also been shown to enhance selectin signaling, however only Fgr binds L-selectin cytodomain. Syk activation of SLP-76 and ADAP results in Btk activation, where signaling becomes PI3Kγ dependent. This catalyzes Vav1 activation and downstream F-actin reorganization that plays a key role in L-selectin clustering. The PLCγ2 activation of p38 and CalDAG-GEFI activate Rap1 and the unfolding of autoinhibited talin-1, which promotes recruitment to PiP_3/2_ and engagement and extension of LFA-1. Force acting on L-selectin catch-bonds transduces the signaling of high affinity LFA-1. Whereas, engagement of PSGL-1 and L-selectin primes MRP8/14 release. Its binding to TLR4 elicits the extension of LFA-1 and supports deceleration and cell rolling. **(B)** Selectin signaling is synergistic with chemokine signaling via GPCR to induce complete activation of integrin. CXCR1/2 ligation by fMLP and IL-8 elicit the dissociation of Gα from Gβγ subunits of G proteins resulting in PI3Kγ activation and PLCβ2/3, a convergence point between CXCR ligation and selectin signaling pathways. F-actin reorganization induced by PI3Kγ also results in transition of PiP_2_ to PiP_3_, which has a higher binding efficiency for Kindlin-3 and talin-1. DAG then activates PKC. Additionally, PLCβ2/3 splits PiP_2_ into IP_3_ and DAG. IP_3_ then binds IP_3_R on the ER to activate calciosome release. The gradient of intracellular calcium and activation of PKC catalyze activation of CalDAG-GEFI and Rap1 mediated integrin activation. This is a second convergence point between CXCR ligation and selectin signaling to activate LFA-1 through talin-1 recruitment. Calcium influx via Orai1 CRAC channels at focal sites of adhesion elicits the release of ER Ca^2+^ stores, which precipitates STIM1 association with the ER. This synergy between CRAC and the ER at the inflammatory synapse represents a positive feedback loop to enhance local calcium entry and the activation of additional LFA-1. **(C)** Tensile force acting on LFA-1/ICAM-1 provides for mechanotransduction of local calcium entry through Orai1 CRAC. Force acting on high affinity LFA-1 transduces from outside-in Kindlin-3 engagement. A conformational shift in the high affinity LFA-1 β_2_-tail exposes the Kindlin-3 binding domain. RACK-1 and Kindlin-3 both localize to the plasma membrane through its engagement with PiP_2/3_ to promote clustering of high affinity LFA-1. RACK1 may be a physical link between Kindlin-3, clusters of high affinity LFA-1, and TRPC3/IP_3_R/STIM1/Orai1; thereby completing a circuit to transduce force mediated calcium entry. RACK1 can activate PKC and CalDAG-GEFI and Rap1 providing a means of crosstalk between integrin outside-in and GPCR inside-out signaling.

Neutrophil rolling in humans is primarily mediated by L-selectin and PSGL-1 on the neutrophil surface and P-selectin and E-selectin on inflamed endothelium (34, 35). The minimum recognition unit of selectins is the tetrasaccharide sialyl Lewis^x^ (sLe^x^), a sialic acid α2-3 linked to galactose anchored by a β1-4 linked N-acetylglucosamine bearing a α1-3 linked fucose (36). sLe^x^ is expressed on glycosphingolipids (GSL), O-glycans of PSGL-1, and N-glycans of L-selectin (Figure 1B) (35, 37). In human's enzyme fucosyltransferases 4, 7, and 9, as well as sialyltransferase ST3-Gal-IV, are required to assemble sLe^x^ on N- and O-linked glycans, and GSL (38–40). Recent studies employed CRISPR-Cas9 gene editing to truncate each of the commonly expressing glycan types, reveal that O-glycans are responsible for leukocyte capture and initiation of rolling while N-glycans and GSL stabilize slow cell rolling and the transition to arrest (41, 42). Extracellular PSGL-1 is decorated in serine and threonine residues that are glycosylated to primarily bear fucosylated O-glycans capped with sLe^x^ that are capable of binding the calcium ion present within the lectin domain of all three selectins (Figure 1B) (43). Human L-selectin is decorated with N-glycans capped with sLe^x^, which enable recognition by E-selectin (44, 45). This data implicates PSGL-1 as a ligand associated with selectin mediated capture and slow rolling, while L-selectin functions as a mechanosignaling ligand of E-selectin on inflamed endothelium. However, a number of studies using transgenic mice deficient in selectins or their ligands indicate that rolling via PSGL-1 is sufficient to mechanosignal integrin activation. One key difference is that fucosyltransferase 9 plays a key role in human, but not mouse neutrophil/E-selectin interactions (39, 40). This indicates a major difference in E-selectin ligands and their potential to signal, specifically that L-selectin in mice is not an E-selectin binding partner. Interestingly, while L-selectin is not an E-selectin binding partner in mouse, both E-selectin ligand-1 and CD44 play critical roles during murine neutrophil rolling and transition to arrest (46). Studies in mice genetic knockouts indicate that ESL-1 cooperates with PSGL-1 to maintain myeloid homeostasis and initiate neutrophil recruitment, but it is PSGL-1 that does the heavy lifting when it comes to integrin activation and slow rolling. In fact, ESL-1 is the predominant E-selectin ligand used by immature hematopoietic progenitors to home to the bone marrow. As myeloid maturation occurs a functional shift in selectin ligands from ESL-1 to PSGL-1 reduces the importance of ESL-1 in selectin signaling (47). Despite L-selectin not being a functional E-selectin ligand, inhibition of L-selectin binding in mice inhibits rolling, which was largely attributed to a loss of neutrophil-neutrophil mediated secondary capture that is L-selectin/PSGL-1 dependent (48). Further highlighting the function of L-selectin in selectin ligand outside-in signaling in mouse neutrophils, Stadtmann et al. reported that PSGL-1 ligation under shear flow precipitates membrane co-localization of PSGL-1 and L-selectin, which in turn elicits outside-in signaling of LFA-1 activation (49). One important component of L-selectin signaling is the multiple intracellular binding sites on the cytosolic domain of L-selectin for binding to tyrosine kinases and other downstream activators such as PLCγ2 (Figure 2A). The colocalization between PSGL-1 and L-selectin in mouse is induced by CD44 on the cell body engaging with E-selectin, which promotes clustering of PSGL-1 and L-selectin on the neutrophil surface in a p38 dependent manner. This was shown to promote secondary leukocyte tethering and formation of the L-selectin/PSGL-1 signaling complex (46). Murine neutrophils with down regulated L-selectin expression do not form this complex and therefore cannot signal for extension of integrin in the absence of chemokine (49). Thus, even in the absence of direct recognition of L-selectin by E-selectin on mouse neutrophils, L-selectin represents a potent selectin ligand outside-in signaling receptor.

Selectin engagement and mechanosignaling in neutrophils is only partially defined, and while other E-selectin ligands may play a role in capture, tethering, and signaling, we will focus on mechanosignaling through L-selectin engagement. E-selectin preferentially recognizes sLe^x^ on L-selectin and PSGL-1 in humans and signals active transport of adhesion molecules, leading to rapid cell arrest in shear flow (45). Binding of E-selectin promotes colocalization of L-selectin and PSGL-1 into membrane clusters on microvilli, an event that temporally correlates with MAPK phosphorylation and focal clustering of high-affinity CD18 (50). Genetic deletion of L-selectin in mice results in loss of phosphorylation of Akt, Syk, and phospholipase C (PLC) γ2, indicating these signaling molecules are downstream of the PSGL-1/L-selectin signaling complex (Figure 2A) (49). PSGL-1 engagement during rolling in inflamed mouse vasculature involves receptor clustering within lipid rafts that recruits membrane FcRγ and cytosolic DAP-12 (Figure 2A). Subsequent phosphorylation by Fgr then recruits Syk (51). The signaling pathway downstream of Syk includes SLP-76 and Bruton tyrosine kinase (Btk), which bifurcates the activation of PLCγ2 and phosphoinositide-3 kinase (PI3K) gamma-dependent pathways (51–53). Ligation of L-selectin and PSGL-1 enhances activation of PI3K, which acts on the effector Vav1 and results in F-actin assembly and downstream L-selectin reorganization and clustering, facilitating a feedback loop that amplifies signaling via L-selectin (54). It is known that PI3K activation results in cytoskeletal changes following leukocyte rolling, however its direct role in mechanosignaling via L-selectin outside of inducing clustering is unknown. A second pathway involving PLCγ2 activates downstream CalDAG-GEFI and p38 MAPK resulting in Rap1a activation (55). Rap1 then facilitates integrin extension and activation by recruiting Talin-1, and potentially Kindlin-3, which in turn allosterically reorients the integrin cytoplasmic domain (56). Talin-1 is an adaptor molecule that binds to the β_2_-integrin cytodomain to initiate extension and the high-affinity state, as well as providing a cytoskeletal anchor for vinculin (12, 56, 57). Integrin activation and clustering via talin-1 facilitates its binding to dimerized ICAM-1, this effectively prolongs the lifetime of LFA-1 bonds by ~10-fold and potentially enhances the association of adaptor molecule recruitment (27). Kindlin-3 is a similar adaptor protein to talin-1 that plays a central role in LFA-1 clustering and is critical for regulation of integrin off-rates (12, 58, 59).

It is well-established that mechanosignaling through L-selectin induces extension of β_2_-integrin, while the mechanism for activation of high-affinity β_2_-integrin independent of chemokine mediated inside-out signaling remains a point of contention. A recent discovery highlights a central role for MRP8/14 (also known as S100A8/S100A9 or calprotectin) as a signaling pathway that elicits extension of β_2_-integrin but not necessarily activation of the high affinity state (60). MRP8/14 is secreted by neutrophils during rolling on E-selectin where it then binds toll-like receptor 4 (TLR4) and signals in an autocrine manner activation of Rap1 and LFA-1 extension (13, 60). Ligation of PSGL-1 was sufficient to elicit release of MRP8/14 and induce extension of β_2_-integrin in a TLR4 dependent manner, whereas clustered L-selectin delivers a distinct signal that activates high affinity β_2_-integrin (13). Thus, bond tension and clustering of L-selectin cooperates with MRP8/14/TLR4 in priming LFA-1 extension, but a distinct signal is necessary for LFA-1 to achieve a high affinity state. Shear-resistant arrest is mediated in human neutrophils through clustered E-selectin/L-selectin bonds via signaling of high-affinity β_2_-integrin. The signaling pathway underlying MRP8/14 and TLR4 activation of Rap1 may involve PLCγ2, but this remains ill defined. Additionally, TLR4 has been shown to drive neutrophil aging which brings up a key point in neutrophil signaling, specifically dependence of senility in altering neutrophil receptor expression (61). Aged neutrophils show an enhanced expression of LFA-1, Mac-1, and CD44 and a decreased expression of L-selectin, however whether these receptor expression changes can be induced by MRP8/14 ligation of TLR4 remains unknown (62–64). Enhanced integrin expression translates to a more efficient shear-resistant recruitment of neutrophils under LPS challenge and implicates aged neutrophils as the first line of defense (65).

## Selectin-sLe^x^ bond mechanics

Hydrodynamic drag force acting on flowing neutrophils induces catch bond formation between E-selectin and L-selectin. The mechanics of selectin-sLe^x^ bonds and how they transduce outside-in signals via Src and Lck kinases are active areas of investigation (53, 66). A logical question is how catch-bond behavior of selectins dictates their capacity to engage in selectin ligand outside-in signaling. Neutrophil selectin binding kinetics have been studied using a variety of techniques including, atomic force microscopy, bioforce probes, surface plasmon resonance, and parallel plate flow channels (3, 67). Selectin bonds exhibit a triphasic adhesive response that is denoted slip-catch-slip bonds (68, 69). Slip bonds exhibit a shortened lifetime as the tensile force acting on them is increased. Catch bonds on the other hand increase lifetime as applied tensile force is increased. Neutrophils bound via PSGL-1 by P-selectin at low wall shear stress (<0.3 dynes/cm^2^) capture sporadically, dissociate frequently, and roll with high velocity (70). In contrast, higher shear stress (between 0.3 and 1 dynes/cm^2^) promotes more efficient capture and steady rolling at constant velocity, indicative of selectin catch bond behavior (69, 71–73). As wall shear stresses exceeds 1 dynes/cm^2^, slip bond behavior is again observed. An experiment that begins to explain the mechanism of catch-bond behavior was application of Rivipansel to antagonize L-selectin/E-selectin bonds, along with selectin ligand outside-in mechanosignaling of high affinity LFA-1 (13). Rivipansel is a rationally designed pan-selectin inhibitor that mimics the sLe^x^ tetrasacharide structure and an extended sulfate domain recognized by all three selectins lectin domain and in clinical trials for treatment of vaso-occlusive crisis in Sickle Cell disease (13, 74, 75). We recently reported that treatment with Rivipansel, blocks catch-bond formation between E-selectin and L-selectin and effectively inhibits selectin ligand outside-in signaling of integrin activation and neutrophil rolling to arrest (13). A structural explanation that begins to shed light on selectin catch-bond behavior was derived from co-crystallization between E-selectin and sLe^x^ that was imaged by small-angle X-ray scattering and modeled with molecular dynamics simulation. This analysis predicted that E-selectin bound to sLe^x^ under force caused an opening in the angle between the lectin and EGF domains from ~120 to ~141° (Figure 1B) (76). This rotation facilitates more efficient engagement of sLe^x^ expressing ligands by E-selectin resulting in an increase in bond strength and resistance to shear flow, indicative of a catch bond. Molecular dynamics simulations of the lectin-EGF domain angle between P-selectin bound with sLe^x^ is predicted to be ~114.6°, similar to the E-selectin/sLe^x^ angle. When tensile force is applied to this complex the interdomain angle opens to an extended conformation of ~140° allowing it to align in the direction of force application resulting in an enhanced off rate (43, 77). This catch-bond strengthening phenomenon has not been reported for L-selectin bound to sLe^x^, but it is highly likely given the homology in lectin structure between the three selectins. A second mechanism that accounts for catch-bond behavior is when force induced dissociation of ligand results in rebinding by the lectin headpiece as the angle between the lectin-EGF domain increases (Figure 1B) (77). Bond lifetime is prolonged through multiple dissociations required for complete ligand detachment. Another model, known as the allosteric model, involves reorientation of the crystal structure of P-selectin when ligand binding elicits a shift in residues 83–89 in the lectin domain, thereby augmenting the binding surface and increasing the affinity between the lectin domain and sLe^x^ (78). Swapping the alanine 28 in the lectin domain with a bulky histidine to effectively open the 83–89 loop, resulted in decreased dissociation constant and slower rolling velocity on a substrate of PSGL-1 (78). It is noteworthy that the slower rolling was not observed at low shear stress regimes, corroborating the slip to catch transition induced at the higher shear stress. Taken together, modeling, and experimentation have shown that tensile force of sufficient magnitude and applied at defined rate can elicit an allosteric shift in selectins that in turn influence the strength and lifetime during bond formation with sLe^x^ presenting ligands. LFA-1 bonds also convert from a low or intermediate affinity state to a high affinity state during rolling at low shear stresses within the catch-bond regime between 0.5 and 2 dyne/cm^2^. This was first demonstrated by neutrophil capture on a substrate co-expressing E-selectin-IgG and high affinity β_2_-integrin reporter mAb24 (13). However, a different study reported that the majority of neutrophils sheared in microfluidic channels at 6 dynes/cm^2^, outside the catch-bond regime, did not activate and arrest on a substrate presenting mAb24, but bound efficiently on a substrate presenting the extension reporting antibody KIM127 (30). Taken together the data indicate that, while catch-bond formation via E-selectin/L-selectin engagement provides a distinct signal to activate high affinity LFA-1 and neutrophil arrest. This elucidates the importance of hydrodynamics in selectin-sLe^x^ catch-bond mechanics to provide a force sensitive mechanism for signaling optimum recruitment at appropriate shear stress.

## Signaling between selectins and GPCR activation converges to activate LFA-1

So far, we have only focused on integrin activation via selectins, it is important to note that GPCR inside-out signaling is capable of transitioning LFA-1 from a low to high affinity state. This begs the question; how does selectin ligand outside-in signaling cooperate with inside-out signaling generated by CXCR1 and CXCR2 engagement by chemokine? While the role of GPCR signaling is well-reviewed elsewhere (79, 80), here we focus on how signaling facilitates LFA-1 mediated neutrophil arrest and a migratory phenotype (Figure 2B). Chemokine stimulation of GPCR in neutrophils results in stimulation of G proteins, separating Gα from the Gβγ subunits triggering PLC-β and PI3Kγ activation. PLC-β cleaves phosphatidylinositol 4,5 biphosphate (PIP_2_) into diacylglycerol (DAG) and inositol-1,4,5 triphosphate (IP_3_) (18, 81). IP_3_ then binds IP_3_ receptor (IP_3_R) localized on the membrane of the endoplasmic reticulum (ER) triggering cytosolic release of calcium (82, 83). The release of calcium within the ER is sensed by stromal interaction molecule 1 (STIM1), which functions to localize the ER to the primary calcium release activated calcium (CRAC) channel, Orai1 on the neutrophil membrane (Figure 2B) (83). Orai1 forms a hexamer that regulates influx of extracellular calcium (22, 84). PI3Kγ signals downstream of GPCR signaling by catalyzing PiP_2_ conversion to PiP_3_ and its association with the membrane near the integrin cytoplasmic domain. PiP_3_ functions to recruit Kindlin-3, Skap2, and other PH domain interacting molecules that are necessary for integrin transition from extension to high affinity more efficiently than PiP_2_ (14, 85, 86). PI3Kγ represents a convergence point between selectin ligand outside-in signaling and inside-out GPCR signaling pathways, and its activity cooperatively regulates activation of integrin. Each signaling pathway elicits a calcium influx, selectins through TRPC channels and chemokine through Orai1, and both pathways converge upon Rap1 dependent activation of integrin. It is widely known that high concentrations of chemokine can promote activation of LFA-1 and the onset of neutrophil deceleration and arrest. It appears that nature has designed a cooperative system by which very low levels of chemokine signaling can superpose with selectin catch-bond dependent signaling to amplify the response and likelihood of recruitment of surveilling neutrophils, perhaps most relevant in skin where E-selectin is expressed at low levels (14, 15). While there is lots of evidence supporting L-selectin and CXCR1/2 cooperativity in neutrophils, there has been no synergy observed between L-selectin and CCR7 signaling for enhancing LFA-1 activation in lymphocytes.

## LFA-1 is requisite for neutrophil arrest

LFA-1 and Mac-1 are both involved in the transition from arrest to a migratory phenotype. However, it appears that the sequence of adhesive events is important for the precise synchronization of transendothelial migration. LFA-1 bonds function to initiate neutrophil arrest, while Mac-1 bonds provide migratory traction. Bond number and strength dictate the adhesive lifetime and translates to the amount of force that is transmitted across the membrane. Forces transmitted via LFA-1 and Mac-1 bonds in neutrophils are in part a function of their respective ligands, ICAM-1 and ICAM-2 are the main ligands on inflamed endothelium bound by activated LFA-1, while Mac-1 primarily binds to RAGE and JAM-C (87). It is noteworthy that LFA-1 can also bind to JAM-A and JAM-C, while Mac-1 recognizes the Ig-domain 3 of ICAM-1, albeit with lesser bond strength (19, 80, 88). Direct measurements of adhesion efficiency and rupture force for Mac-1 and LFA-1 bonds locked into a high or low affinity state were performed using atomic force microscopy (AFM) targeting the slip bond regime (87). An AFM tip was functionalized with LFA-1 or Mac-1 locked into specific states via allosteric antibodies or activated via manganese. Bond formation was induced by bringing this tip into contact with a surface of counter ligands ICAM-1, ICAM-2, RAGE, JAM-A, or JAM-C and then retracted at various rates. Deflection of the cantilever, as measured by a deflection of a laser beam reflected off the back of the cantilever, brought to light distinct features of bond rupture force and lifetime. The differences between mean rupture force of high affinity and low affinity LFA-1 was most pronounced when bound to ICAM-1 (56.1 ± 4.1 pN), ICAM-2 (37.7 ± 2.0 pN), JAM-A (37.4 ± 4.3 pN), and JAM-C (34.0 ± 5.9 pN) (87). The difference between high affinity and low affinity Mac-1 was most pronounced when bound to JAM-C (32.0 ± 2.8 pN) and RAGE (25.2 ± 4.2 pN) (87). When activated to high affinity, LFA-1/ICAM-1 and Mac-1/JAM-C bonds show the greatest strength. This correlates well with the observed function in the adhesion cascade of each integrin subunit, specifically LFA-1 mediates shear resistant cell arrest, while Mac-1 functions primarily in cell migration. It is important to note that the differential spatial localization of LFA-1 (on microvilli) and Mac-1 (on microvilli and cell body) on the neutrophil surface may result in each bond experiencing a distinct force regime during arrest in shear flow. This in turn can influence catch-bond behavior and result in different functional roles for each subunit during ligand binding.

Given that the magnitude of shear stress dictates the efficiency and lifetime of adhesion (89), it is critical to review how molecular mechanics are regulated and activated compared to quiescent neutrophils. Evans et al., was the first to measure off-rates and bond lifetimes between LFA-1 and ICAM-1 at the single integrin scale utilizing a bioforce probe (90). Activated LFA-1 possesses persistent mechanical strength exceeding 20 pN per bonds with lifetimes on the order of ~1 s when tensile force is applied at rates of ~10 pN/s. When force was ramped between ~10 and 1,000 pN/s it was observed that unbinding increased exponentially, indicating that LFA-1 bond lifetime is highly sensitive to force application. When locked in a high affinity state in Mn^2+^ enriched buffer, LFA-1 lifetime decreased to ~3 ms at a bond strength of ~64 pN (90). When these curves were extrapolated to zero force, bond lifetime increased to ~2 min corresponding to an off rate of ~0.05/s. As force ramps were increased to very high levels exceeding 7,000 pN/s, the force sensitivity of the off rates between LFA-1 and ICAM-1 disappeared, suggesting that large forces induce a change in molecular configuration of the complex (90). Locking recombinant LFA-1 at high affinity with Mn^2+^, or for native LFA-1 on neutrophils with allosteric activating antibody 327C or stimulation with IL-8, and testing bond formation with recombinant dimeric ICAM-1 revealed nearly identical changes in off-rates as force was ramped (91). Taken together, these studies indicate that a conformational switch elicited by either inside-out or selectin ligand mediated outside-in signaling results in LFA-1 heterodimers binding in tandem with domains 1–2 of parallel ICAM-1 molecules to establish long-lived bond formation that supports neutrophil firm arrest (Figure 2C).

Using the bioforce probe technique to ligate single LFA-1 molecules and measure bond kinetics it was subsequently reported that LFA-1/ICAM-1 bonds experience catch-slip bond behavior (92). Three states and corresponding distinct off-rates were identified at defined force profiles: At zero force, allosteric activation of LFA-1 to a high-affinity state elicited the most efficient binding. However, when force was ramped to ~10–15 pN on LFA-1/ICAM-1 bonds, lifetime increased to a maximum, revealing catch-bond behavior dependent upon the high affinity state (92). Beyond a threshold level of force, bond lifetimes monotonically decreased, indicative of slip bond behavior. Moreover, when LFA-1 was locked into a low affinity or extended conformation catch bonds were not detected, rather bond lifetime decreased monotonically. A structural model was proposed whereby pulling on extended LFA-1 anchored to the cytoskeleton elicits a shift in the α7 helix, thereby exposing the MIDAS domain and a shift to high affinity LFA-1 (Figure 1C). A structural model by which force stabilizes the high affinity conformation was proposed that involves movement of the α7 helix in the α_L_-I domain linking to an intracellular loop that shifts orientation of the adjacent AMIDAS domain in the β_2_-I domain (93). This was experimentally supported utilizing the antagonist XVA143 that binds internally to a site between the α_7_ helix and the AMIDAS domain on the β-I domain. This effectively blocked catch-bond behavior, reduced ligand binding affinity under force, and decreased bond lifetimes. This reveals the importance of the α_7_ helix and its binding to the intra-ligand AMIDAS domain in forming strong long-lasting catch-bonds (92). However, when force is applied the intra-ligand interaction is enhanced, indicating that force is necessary to precipitate the complete maturation of high affinity LFA-1. This external shift in geometry of β_2_-integrin suggests that uptake of tensile force can reinforce bond strength and lifetime via a shift in the angle of the transmembrane domain. As discussed below, we propose that a shift in transmembrane domain angle with force is responsible for initiating outside-in signaling by transducing a deformation in the Kindlin-3 binding domain within the β_2_-integrin cytoplasmic tail. This in turn may catalyze the association of PiPs to the integrin to enhance adaptor molecule recruitment to the site of high affinity LFA-1.

## Integrin LFA-1 cytoskeletal adaptor proteins function in mechanosignaling

Kindlins are a family of proteins that are highly conserved and function as cytoplasmic adaptor proteins that bridge the cytoskeleton to integrins via their FERM domains. A rare mutation in Kindlin-3 is the culprit in leukocyte adhesion deficiency type-III, a disease characterized by defects in leukocyte and platelet β_1_-, β_2_-, and β_3_-integrin functions (6, 94–97). For LFA-1 to transition from low to intermediate and high affinity, engagement of the cytoplasmic domains by talin-1 and Kindlin-3 are necessary. These adaptor proteins are 4.1/ezrin/radixin/moesin (FERM) domain proteins with four subdomains (F0–3), whose F3 domains are capable of binding one of two specific NPxF/Y domains present on β-integrin cytoplasmic tails (98). Talin-1 also has an extended rod domain that binds actin, indicating a key role in F-actin association and local cytoskeletal rearrangement (56). NMR spectroscopy revealed that the talin-1 rod domain interacts with the F3 subdomain, masking its binding domain. This autoinhibition is disrupted by PiP engagement allowing the talin-1 F3 domain to bind the proximal NPxF/Y on β-integrin tails and break the salt bridge holding the α-β chains together, thereby initiating the transition to an extended and a high affinity state (99, 100). Kindlin-3 is endowed with a plexstrin homology (PH) domain embedded into its F2 subdomain. Kindlin-3 F3 domain binds to the membrane distal NPxF/Y motif on the β-integrin tail (56). Constitutive Kindlin-3 is not autoinhibited nor does it bind to low affinity LFA-1, indicating that its β-integrin binding domain is not exposed until integrin extension occurs. Transgenic mice in which talin-1 is genetically deleted lacks the capacity to both extend or activate high affinity integrin, while Kindlin-3 knockouts retain the capacity for LFA-1 extension, but not activation to high affinity (58). Remarkable was the finding that a 95% knockdown of Kindlin-3 in a mouse model, retained basal levels of integrin function in platelets (101). However, extended bleeding and impaired healing was observed when these mice were exposed to injury and infection. This indicates that a threshold level of Kindlin-3 and talin-1 are necessary to maintain normal function of LFA-1 (101). In fact, Kindlin-3 and talin-1 abundance is sufficient to occupy only ~50% of integrin cytodomain in granulocytes. By comparison, platelets contain twice as much adaptor proteins, this highlights a key difference in LFA-1 activation kinetics compared with GPIIbIIIa that also requires Kindlin3 for function (101). A stoichiometric balance exists between Kindlin-3/talin-1 and integrin in neutrophils, such that diffusion may be a limiting factor in the rate of LFA-1 activation.

Loss of Kindlin-3 function in patients suffering from LAD-III is characterized by suppression of LFA-1 functions, but not VLA-4 under shear conditions in both neutrophils and primary T cells (94). Tensile force, talin-1 and Kindlin-3 are necessary conditions to observe activation of high affinity LFA-1. One potential mode of LFA-1 activation is via talin-1 recruitment to the β-subunit tail thereby catalyzing the extended conformation and recognition of ICAM-1. As fluid drag transmits tensile force to the intermediate affinity bond, molecular deformation exposes the MIDAS and precipitates a transition to the high affinity state. Given that LFA-1 extension is observed to promote the engagement of Kindlin-3 at sites of focal adhesion, it is possible that force transmission on LFA-1 itself catalyzes increased binding of Kindlin-3 (16). However, the precise mechanism by which these adaptor proteins recruit to LFA-1 is ill defined, as is whether they simultaneously reside on a single LFA-1 cytodomain. It has been suggested that Kindlin-2 and talin-1 are capable of simultaneously binding a single β_2_-integrin tail, and due to the homology between Kindlin-2 and talin-1 F3 domain it is highly likely that Kindlin-3 can also bind to the integrin tail simultaneously with talin-1 (102). Adaptor protein binding occurs following phosphorylation of the tyrosine in their respective binding sites via Src family kinases. Binding is modulated by another key TTT phosphorylation site between the two binding regimes (58). Kindlins, filamin, 14-3-3 and other proteins can bind this domain and can affect the order in which binding to the other NPxF motifs occurs (103, 104). Kinases provide spatiotemporal regulation of integrin activation, but more research is required to elucidate its precise role in mechanosignaling. Given that talin-1 binding is retained in Kindlin-3 knockouts, a prevailing theory is that these adaptor proteins may serve as co-activators by removing potential competitive binding proteins such as 14-3-3 protein (103). An additional mechanism is via talin-1 induced extension to expose the binding site of Kindlin-3 on the integrin tail allowing it to then function as a mechanosensitive clutch. Experimental data indicates that talin-1 and Kindlin-3 play independent roles during signaling of neutrophil arrest and migration. Utilizing neutrophil-like HL-60 cells to knockout talin-1 or Kindlin-3, activated LFA-1 bonds under tensile force catalyzed calcium influx through the CRAC channel Orai1 only in the presence of Kindlin-3. The presence of talin-1 and absence of Kindlin-3 was insufficient to link LFA-1 to Orai1 and induce calcium influx (16). These data provide insight on mechanotransduction through LFA-1 under shear force conditions, which involves assembly of a complex via Kindlin-3-β_2_-integrin cytodomain and Orai1 to complete a circuit whereby force induces calcium flux.

Kindlin-3 association with LFA-1 is necessary for the rapid clustering of LFA-1, but it is unlikely to function as a scaffold protein in this process since it has only one binding site for the β_2_-integrin tail, unless Kindlin-3 is capable of complexing other Kindlin-3. Another adaptor protein that can enhance LFA-1 clustering is receptor of activated protein C kinase 1 (RACK1) (Figure 2C). RACK1 is a seven bladed propeller protein that can bind multiple Kindlin-3 with its domains 5 to 7. Kindlin-3 binds RACK1 through its PH domain and in cells with the PH domain deleted, LFA-1 clustering is inhibited (59). However, Kindlin-3 PH domains play a key role in binding numerous proteins such as SKAP2 or PiP_2_. Thus, knockout of the PH domain may suppress Kindlin-3 migration to the LFA-1 tail domains, independent of RACK1 (80, 86). Despite this, it is noteworthy that immunoprecipitation of a ternary complex between β_2_-integrin tail, RACK1, and Kindlin-3 is intact even when the Kindlin-3 F3 domain is genetically deleted (59). Given that RACK1 itself does not activate adaptor proteins, it may function as a chaperone for other adaptor proteins to bind the integrin cytodomain. RACK1 has also been shown to bind focal adhesion kinases (FAK) and Src via propeller domain 2 to promote IGF-1R receptor association with integrin, and in a similar way may induce LFA-1 clustering by promoting Kindlin-3 association under tensile bond force (105). RACK1 structure shares a similar homology to Gβ subunit and has been shown to form a heterodimer with it (106). While it is clear that RACK1 plays a role in membrane clustering of LFA-1, whether that is due to aggregation of Kindlin-3 bound to LFA-1 or by promoting the assembly of additional adaptors requires further study. Kindin-3 induced LFA-1 clustering correlates with enhanced calcium signaling, yet the complete signaling circuit has yet to be elucidated (16).

## Cytoskeletal activation and motile function regulated by local Ca^2+^ influx

Hydrodynamic force acting on LFA-1 and Mac-1 regulates calcium entry, kinase activation, and cytoskeletal protein recruitment all of which are necessary to achieve a migratory state (15, 107–109). We propose that LFA-1 functions not only as a breaking mechanism to achieve neutrophil arrest, but also in the mechanotransduction signals delivered through focal sites of adhesive traction that oppose shear force gradients present on the endothelial surface (Figure 2C). Cooperativity between selectin engagement and chemokine binding of GPCRs activate the transition of LFA-1 from low to high affinity resulting in deceleration of the cell that occurs on the order of seconds (12). Neutrophil deceleration and arrest trigger a concomitant rise in intracellular calcium detected within seconds and which precipitates cell shape change and polarization within minutes (15). Coordination in signaling rolling to arrest and to a migratory state is interrupted by inhibiting CRAC channels with pharmacological inhibitors, or genetic deletions that alter calcium flux (22, 110). The precise number of LFA-1 receptors associated with signaling calcium flux is unknown, however, once sufficient numbers of LFA-1 transition to high affinity bonds (on the order of ~100 receptors) within ~2–3 submicron focal microclusters, local calcium entry via Orai1 is initiated promoting the coalescence of LFA-1 into micron sized macroclusters (16). This feedback loop between enhanced LFA-1 clustering and Orai1 mediated calcium entry results in a large local transient burst of intracellular calcium that is required to promote organization of high-affinity integrin within focal adhesions. This is in contrast to GPCR that are distributed around the neutrophil within microvilli, and upon ligand binding provide an inside-out signal that is more globally dispersed within the cell volume. This implicates integrin mediated calcium signaling as a central regulator of neutrophil migratory function beyond firm arrest (Figure 2C). Remarkably, RACK1 has been shown to regulate IP_3_R function in a manner dependent on TRPC3 that in turn promotes calcium release (111). Once calcium has been released through IP_3_R activation via RACK1, IP_3_R associates with activated STIM1 and subsequently binds Orai1 (111). Further, TRPC3 deletion in HELA and Hek cells, abrogates the association between Orai1 and IP_3_R (111). While this has yet to be shown in primary human neutrophils, these data highlight the potential for high affinity LFA-1 bonds under force to catalyze association of a complex composed of Kindlin-3/RACK1/TRPC3/IP_3_R/STIM1/Orai1 that effectively directs calcium influx and release of ER stores within focal adhesions in a manner that orients cytoskeletal force generation and neutrophil polarization (Figure 2C).

A lack of calcium release or entry via CRAC impairs various physiological events in immune cells, implicating calcium as a pivotal secondary messenger (23, 24, 81, 82, 107–109, 112–114). The role of calcium in T cell regulation can provide insight into calcium signaling in neutrophils. Through the use of genetically-encoded calcium indicators it has been shown that T cell interaction with antigen presenting cells *in vivo* results in low levels of local calcium release (115). Local calcium enhances T-cell mechanosignaling within the immune synapse by promoting T cell receptor clustering and the binding of anionic phospholipids within the plasma membrane, similar to how local calcium bursts in neutrophils regulates activation and integrin build-up within the inflammatory synapse at sites of focal adhesions. Furthermore, calcium entry via Orai1 is responsible for T cell homing to lymph nodes and is necessary for high-affinity integrin LFA-1 activation (116). The magnitude of calcium bursts builds over time and function to recruit more LFA-1, which in turn activates additional Orai1 in a feedback loop to promote adhesion and signaling. Once LFA-1 is engaged between the T cell and antigen presenting cell, external calcium concentration rises above cytosolic, lending credence to the theory that co-localization between membrane receptors and CRAC provides a spatially localized signal that is scaled by the surface area of the cluster which dictates its contribution to cell activation. Neutrophils appear to engage in a similar mechanical process in which LFA-1 bond traction provides spatiotemporal cues, but this occurs within seconds as opposed to hours for T cells and serves to synchronize the multistep process leading to transmigration.

LFA-1 bond formation provides a spatial queue, while calcium provides a temporal queue to signal cell shape change and polarization. Localized calcium flux provides a signal to initiate local cytoskeletal reorganization and subsequent cellular motility (Figure 2C). Contractile and protrusion forces created by filamentous actin (F-actin) during cytoskeletal reorganization enables the formation of pseudopods that lead migration and contractile rings that organizes formation of the uropod at the rear that generates traction force (117–119). We propose that local generation of calcium gradients generated by CRAC channels concentrated within sites of focal adhesion provides a signal to catalyze cytoskeletal actin formation and interaction with myosin to drive immune cell motility (119). In T-cells sustained calcium is necessary for continued actin polymerization and microcluster formation within the immunological synapse between the T-cell and antigen presenting cell (120). In neutrophils, deficiency of Wiskott-Aldrich syndrome protein (WASp) results in defects in β_2_-integrin clustering, signaling of calcium flux, and cell motility (117, 121). This implicates F-actin mediated cytoskeletal reorganization in integrin clustering and highlights the importance of calcium signaling in this process. Enhanced calcium signaling promotes additional F-actin polymerization and cell spreading through binding to gelsolin a 6-domain actin binding protein that uses calcium to regulate actin filament assembly (122, 123). Once calcium is bound, gelsolin undergoes a conformational change that exposes its actin binding site, thereby promoting cytoskeletal F-actin assembly (124–126). The asymmetry of front/back actin polymerization may be a consequence of the spatial pattern of integrin mediated calcium entry. F-actin also plays an important role in internalization of CRAC channels, providing a putative mechanism for down regulating extracellular calcium entry as neutrophils prepare to transmigrate at appropriate sites of inflammation (21). This illustrates a key feedback mechanism in which calcium entry and cytoskeletal reorganization provides feedback to organize a migratory phenotype in immune cells.

## Conclusions and perspectives

Neutrophils function as the sentinels of the innate immune system by patrolling miles of vasculature in the microcirculation. To accomplish this critical function, they have evolved adhesive mechanisms that facilitate efficient recruitment at the precise location of tissue insult through the conversion of tensile bond force into biochemical signals. This review provides a scheme by which neutrophil tethering and rolling via selectins leads to integrin activation and shear resistant arrest, a set of mechanosignaling based events necessary for subsequent generation of neutrophil protrusions and diapedesis. The latter process is thought to require a chemotactic gradient that guides neutrophils to the site of tissue insult. In a previous *Frontiers of Immunity* review, we detailed how cytosolic release of Ca^2+^ converges with influx through CRAC to dynamically modulate the number and location of β_2_-integrin bonds, which function to synchronize the transition from rolling to arrest and neutrophil shape polarization necessary for diapedesis (9). Recent studies have lent quantitative insight into the physical mechanisms by which L-selectin and integrin catch-bonds convert shear stress into chemical signals within distinct regions of plasma membrane enriched in kinases, phosphoinositides, and cytosolic adaptors (13, 39, 42, 49, 76). Although the specific mechanism of outside-in mechanosignaling is lacking, experimental evidence and structural models indicate that LFA-1 cytosolic domains directly complex with Kindlin-3 and Orai1 and this is regulated by the magnitude of tensile force. We propose that bond tension at durable sites of focal adhesive contact cause reorientation of the integrin headpiece with ICAM-1 and strengthening of the bond. This concomitantly elicits deformation of the LFA-1 cytodomain, thereby exposing the binding site for the PH domain of Kindlin-3 (16, 87, 96). In this review, we put forth the premise that the conversion of LFA-1 to a high affinity state capable of stable bond formation with ICAM-1 is a gatekeeper of this mechanically sensitive linkage that governs transmembrane Ca^2+^ influx. This in turn, facilitates recognition and binding by Kindlin-3 and talin-1 that leads to engagement with RACK1 and FAK and activation of STIM1/Orai1 channels within the focal region of contact on an arrested neutrophil. This contact-mediated circuit is triggered by tensile force conducted via LFA-1 bonds, promotes the calcium feedback loop to recruit additional high-affinity LFA-1 into macroclusters that serve as a nexus for Rho-GTPase activation and F-actin polymerization at contractile regions through which lamellipodia form (127). At sufficient levels of intracellular calcium, F-actin polymerization links to talin-1 tails that reinforce the binding of vinculin. Shape change and cell migration is then mediated by Mac-1 redistribution and bond formation at the uropod where myosins assist in contractile force generation and actin movement (128). In this manner, high affinity integrin bonds effectively function as tactile sensors of the magnitude and direction of hydrodynamic drag forces. Thus, neutrophils dynamically redistribute focal adhesions in a pattern that directs intracellular calcium flux that orients the major axis of neutrophil polarization and generation of motile force to direct innate immune cells at appropriate sites experiencing inflammation.

## Author contributions

VM wrote the initial draft of the manuscript with the aid of SS. VM and SS both edited the manuscript to its current form. VM designed the figures and SS edited the figures.

### Conflict of interest statement

The authors declare that the research was conducted in the absence of any commercial or financial relationships that could be construed as a potential conflict of interest.
